# Donor Kidney Assessment—Functional Metrics and Biomarkers During Ex Situ Machine Perfusion

**DOI:** 10.1097/TP.0000000000005876

**Published:** 2026-08-19

**Authors:** Karim Bousnina, Karin Boer, Robert J. Porte, Martin J. Hoogduijn, Robert C. Minnee

**Affiliations:** 1 Erasmus MC Transplant Institute, Department of Internal Medicine, Division of Nephrology and Transplantation, Erasmus University Medical Center, Rotterdam, the Netherlands.; 2 Erasmus MC Transplant Institute, Department of Surgery, Division of HPB and Transplant Surgery, Erasmus University Medical Center, Rotterdam, the Netherlands.

## Abstract

The shortage of suitable donor kidneys remains a major challenge in transplantation. To address this shortage, kidneys from circulatory death donors and donors who do not meet the standard criteria are increasingly used. However, such kidneys are associated with poorer posttransplant outcomes and would therefore benefit from robust pretransplant evaluation. Machine perfusion technology enables noninvasive, real-time evaluation of kidney grafts through functional measurements and biomarker analysis in the circulating perfusate and urine. These measurements have the potential to determine kidney graft quality before transplantation and potentially predict posttransplant outcomes. However, reliable evaluation tools are not yet well-established and approximately 18% of donor kidneys are still ultimately discarded based on subjective assessments. Evidence indicates that physiological and biochemical parameters, such as renal blood flow and endocrine function, can be used to assess graft quality objectively. Additional perfusate biomarkers identified include immune mediators, markers of cellular stress, injury and death, metabolic biomarkers, and imaging-based assessments. This review highlights a diverse range of perfusion parameters, as well as urinary and perfusate biomarkers that may give valuable information about donor kidney quality and possibly associate with posttransplant outcomes. Nonetheless, most markers have not yet been clinically validated, and their predictive value for posttransplant outcomes remains uncertain. Standardized study designs and integration of multiple quality assessment parameters are essential to advance donor kidney assessment.

## METHODOLOGY

Relevant literature for this narrative review was identified through a PubMed search. Search details appear in the methods box below.

**Table:** 

Search terms	Machine perfusion, ex vivo perfusion, ex situ perfusion, kidney, renal, perfusate, biomarker, metabolite, cytokine, protein, quality assessment, function, imaging
Time window	2015–2025
Inclusion	Studies investigating perfusate biomarkers and other techniques to assess kidney function and/or quality pretransplant or predict posttransplant outcomes
	Studies with human kidneys (transplanted or discarded); animal models only included where human data were unavailable for a given topic
	Studies reporting posttransplant outcome data
Exclusion	Studies with no machine-perfused kidneys
	Studies exclusively reporting well-established biomarkers (neutrophil gelatinase-associated lipocalin, glutathione S-transferase, lactate dehydrogenase), which were outside the scope of this review focused on emerging assessment techniques
	Case reports

## INTRODUCTION

The global burden of chronic kidney disease continues to rise,^1^ while the shortage of suitable kidney donors persists. Despite relatively stable kidney transplant waitlist numbers over the past decade in both the Eurotransplant region and the United States, an estimated 6%–10% of transplant candidates are removed from the waitlist annually because their health deteriorates to the point that transplantation is no longer possible, or they die while waiting.^2,3^ At the same time, the number of deceased donor kidneys increased over the past 10 y.^2,3^ Still, the persistent shortage of suitable organs has led to the increased utilization of circulatory death donors and expansion of donor eligibility criteria. However, kidneys from such donors are more susceptible to ischemic injury and are associated with suboptimal short- and long-term outcomes.^4^ If the perceived risk of transplanting these kidneys outweighs their anticipated benefits, they will not be used for transplantation. On average, 13%–22% of the deceased donor kidneys in the Eurotransplant region and United States are not used.^2,3^ With approximately 73% of all transplanted kidneys procured from deceased donors,^2,3^ accurate pretransplant assessment of donor kidney quality and function is therefore essential to increase the number of transplantations while ensuring optimal outcomes.

Evaluation during hypothermic machine perfusion (HMP) and normothermic machine perfusion (NMP) provides a strategy to address these challenges. Functional assessment of the kidney and analysis of the perfusate and urine during machine perfusion can be used to quantify potential biomarkers, objectively assess kidney graft quality and potentially aid in predicting short- and long-term outcomes. In this narrative review, we discuss functional metrics and biomarkers during ex situ kidney machine perfusion that may predict posttransplant outcomes, including delayed graft function (DGF) and acute rejection, with a focus on emerging indicators (Figure 1).

**FIGURE 1.**
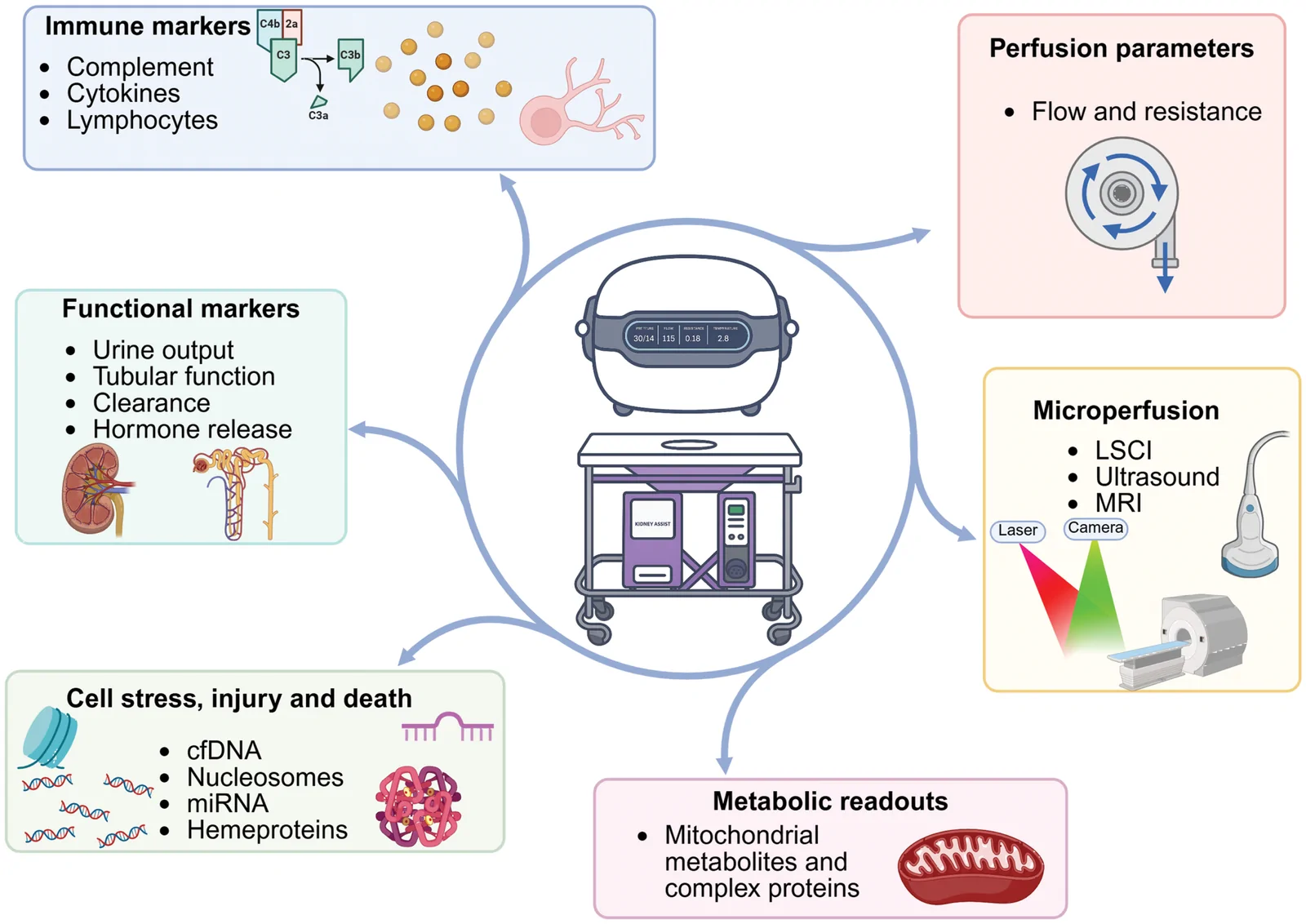
Graphical overview of the biomarkers and other assessment methods during kidney machine perfusion. Measurements can be for either hypothermic machine perfusion (HMP) or normothermic machine perfusion (NMP) and reflect various physiologic aspects of the kidneys. cfDNA, cell-free DNA; LSCI, laser speckle contrast imaging; miRNA, microRNA. Created using BioRender.

## PERFUSION PARAMETERS

Machine perfusion provides real-time perfusion parameter data, including perfusion pressure, flow, and renal resistance (RR), which may be informative of graft quality. Increased resistance or the inability to maintain stable flow can indicate endothelial dysfunction, vasoconstriction, microvascular obstruction, or tissue edema, which all have major implications for graft function. Consequently, hemodynamic parameters during machine perfusion offer a potential marker for kidney graft quality.^5^

### Hemodynamic Parameters

Ahmadi et al^6^ reported that a decline in renal blood flow (RBF) was accompanied by reduced urine production and increased levels of damage-associated neutrophil gelatinase-associated lipocalin (NGAL) during NMP of discarded human kidneys. Notably, an earlier study revealed that RBF at 1-h HMP was independently and inversely associated with DGF.^5^ The authors also reported that RBF at all time points during HMP was positively correlated with estimated glomerular filtration rate (eGFR) at 6 mo posttransplant.

Since increased perfusion flow rates can also result from higher perfusion pressures, the perfusion flow index (PFI) was introduced, wherein the flow rate is corrected for pressure.^7^ It was demonstrated that a higher PFI during HMP was an independent predictor of graft survival, and PFI categories (low, medium, and high) correlated with eGFR up to 4 y posttransplant.

Because RBF is inversely related to vascular resistance, RR may also provide a valuable insight into vascular health during machine perfusion. During HMP, RR progressively decreases whereas RBF increases.^5,8^ However, RR is only significantly higher for discarded kidneys compared with transplanted kidneys and does not associate with posttransplant outcomes.^5^ In contrast, other studies reported that RR during HMP is associated with early graft function.^8,9^ Both relative RR decrease^8^ and absolute RR values, as well as the time required to reach favorable thresholds, are associated with immediate graft function (IGF), whereas persistently high RR correlated with DGF.^9^ Moreover, relative RR decrease correlates with serum creatinine and eGFR on the first 2 d posttransplant.^8^ Importantly, Bissolati et al^9^ reported that conventional Karpinski histological analysis of time-zero biopsies did not predict posttransplant outcomes, highlighting the potential added value of functional perfusion parameters. Dumbill et al^10^ also observed numerical differences in RR during NMP between kidneys with DGF and those with immediate function.

### Microperfusion

One of the reasons to discard a donor kidney is poor in vivo perfusion before procurement. The kidney cortex contains the nephrons, which require sufficient oxygen to prevent injury. Therefore, proper perfusion of the cortex is a key quality assessment measure. Various imaging techniques have been investigated as a tool to monitor renal perfusion during machine perfusion.

#### Laser Speckle Contrast Imaging

Laser speckle contrast imaging (LSCI) has been investigated as a tool to monitor renal cortical microperfusion. Heeman et al^11^ demonstrated that LSCI could detect increased cortical microperfusion during 1-h NMP of porcine kidneys, with a good correlation between laser speckle perfusion units and RBF. Similar findings were reported by Fang et al,^12^ who reported a linear correlation between LSCI flux and RBF. These authors also demonstrated that LSCI can be used to visualize and semiquantify ischemic lesions upon renal artery occlusion. Its clinical relevance remains to be validated.

#### Ultrasound

Two studies reported the use of ultrasound and power Doppler (PD) ultrasound imaging in kidney quality evaluation during NMP.^13,14^ Porcine kidneys were divided into 2 groups based on creatinine clearance and oxygen consumption during 2 h NMP. The investigators measured PD metrics, such as vascularization index, flow index, and vascularization flow index during NMP. The measured PD metrics progressively increased during NMP, indicating improvement of renal perfusion. Notably, PD measurements obtained at 2 h demonstrated the highest diagnostic accuracy for distinguishing between the 2 groups, with higher values in well-functioning kidneys, reflecting more robust perfusion compared with poor functioning kidneys.^13^ Another study used contrast-enhanced ultrasound to measure microvascular perfusion during NMP. The authors reported that improved microvascular perfusion is associated with reduced DNA fragmentation in tissue, a hallmark of apoptosis, and urinary NGAL.^14^ Notably, kidneys with more DNA damage at the start of perfusion displayed worse perfusion in both the cortex and medulla at 2 h. Nonetheless, the effect on clinical outcomes remains unknown, as both studies included porcine and discarded human kidneys.

#### MRI

An MRI-compatible setup has also been used to visualize intrarenal flow distribution in porcine and discarded human kidneys.^15^ These authors observed that perfusion was initially confined to the central region of the kidney, whereas the outer cortex became well perfused after 1–2 h of NMP. Although the kidneys appeared uniformly pink on visual inspection, MRI revealed a markedly heterogeneous perfusion pattern, highlighting that subjective, visual appearance does not necessarily reflect actual perfusion and may therefore not be a reliable assessment tool. Interestingly, although some kidneys exhibited stable flow, the corticomedullary perfusion ratio increased over time, indicating that externally measured flow rates may not accurately reflect intrarenal flow distribution. Nevertheless, apart from a significant decline in fractional excretion of sodium (FENa) over time, glomerular filtration, renal metabolism, and cellular injury remained unchanged, suggesting that variations in flow distribution did not significantly impact overall kidney function.^15^ A study exposing 30 porcine kidneys to 75 min of warm ischemia found that ischemic injury significantly altered regional blood flow distribution during NMP.^16^ Interestingly, RBF measured externally by the machine, reflecting the entire kidney, was only different during the first hour, whereas regional perfusion differences persisted throughout the entire NMP period. Other functional measurements, including urine output, creatinine clearance, and FENa, were also different between both groups.

Overall, these studies demonstrate that hemodynamic measurements during machine perfusion are associated with posttransplant outcomes, including DGF and eGFR (Table 1). A key advantage of these measurements is that they can be obtained continuously and in real-time without the need for tissue or perfusate sampling. However, their interpretation is limited by correlations between different hemodynamic parameters and their dependence on kidney weight (Table 1). Emerging techniques such as LSCI, which enable real-time assessment of cortical microperfusion, may provide additional means of evaluating graft quality. Although these microperfusion-focused approaches offer high-resolution, real-time imaging of renal (cortical) perfusion, they remain exploratory, are not yet standardized, and may be costly to implement (Table 1).

**TABLE 1.** Overview of the parameters and (bio)markers measured during kidney machine perfusion, their clinical relevance, developmental stage, and (dis)advantages

Parameter/(bio)marker	Machine perfusion technique	Clinical relevance	Stage of development	(Dis)advantages
RBF	HMP	Inversely associated with DGF and positive correlation with eGFR^5^	Clinical	Advantages
	NMP	RBF decline is associated with reduced urine production and increased NGAL during perfusion of discarded kidneys^6^	Preclinical	
				Continuous, real-time readout
				Established predictor of DGF in HMP
				No sampling required
				Disadvantages
				Affected by perfusion pressure
				No information on tubular or glomerular function
Perfusion flow index	HMP	Predictor of graft survival and correlates with eGFR^7^	Preclinical	Advantages
				Normalize flow to organ weight—more comparable across organs
				Corrected for pressure-dependent variation in flow rate
				Disadvantages
				Dependent on accurate organ weight measurement
				Only described for DCD kidneys
Renal resistance	HMP	Renal resistance decrease is associated with IGF, serum creatinine and eGFR on the first 2 d posttransplant^8^	Clinical	Advantages
		High renal resistance is correlated with DGF^9^		
	NMP	Numerical difference in renal resistance between DGF and IGF kidneys^10^		
				Continuous, real-time readout
				No sampling required
				Disadvantages
				Sensitive to perfusate viscosity
				Cannot localize resistance (arterial, microvascular, edema)
Microperfusion	NMP	LSCI	Exploratory	Advantages
		Associated with RBF and used to semiquantify ischemic lesions^12^		
		Ultrasound		
		Higher power Doppler metrics reflect better perfusion of well-functioning kidneys^13^		Real-time imaging capability
		MRI		Measure microvascular circulation
		Ischemic injury affects regional perfusion and could be used to identify well-functioning from poorly functioning kidneys^16^		Visualize cortical perfusion
				Disadvantages
				Technique-dependent; limited standardization
				LSCI: limited measurement depth and complex scattering physics
				MRI: difficult and costly to implement in clinical setting
Urine output	NMP	Higher urine output is associated with shorter hospital stay and IGF^10,17^	Clinical	Advantages
				Real-time functional measurement
				Disadvantages
				NMP only
				Affected by perfusion pressure
Proteinuria	NMP	Positive correlation with eGFR at 3 mo posttransplant^10^	Clinical	Advantages
				Real-time readout
				Reflects glomerular barrier integrity
				Disadvantages
				NMP only
FENa	NMP	Well-performing kidneys excrete less sodium compared with poor performers^6^	Clinical	Advantages
				Distinguishes tubular reabsorption from filtration
				Disadvantages
				Laboratory-based testing
				Requires urine and perfusate sampling
				NMP only
Clearance	NMP	Better grafts showed improved creatinine clearance during NMP^6,18^	Preclinical	Advantages
		Inulin clearance during NMP could be used to classify fully functional, limited function, and nonfunctional kidneys^19^		Gold-standard GFR measure
				Most directly comparable measure to in vivo glomerular filtration
				Disadvantages
				Laboratory-based; serial sampling required
				Not feasible during HMP
Hormones	NMP	More urodilatin during NMP of IGF kidneys and inversely correlated with DGF duration^20^	Exploratory	Advantages
		Positive correlation between perfusate EPO and day 7 serum creatinine^20^		May reflect cell-specific viability
				Disadvantages
				Laboratory-based testing
				No validated thresholds in machine perfusion context
Cytokines and complement	HMP	Perfusate C3 is inversely associated with 12-mo eGFR^21^	Exploratory	Advantages
	NMP	Increased perfusate IL-18 is associated with DGF^10^		
		Perfusate C3b and C5b-9 are associated with DGF and an increased risk of prolonged DGF^22^		
		Increased perfusate C5b-9 was associated with impaired creatinine decline during NMP^23^		
				Multiplex panels allow broad simultaneous profiling
				Provides mechanistic insight in inflammatory injury
				Disadvantages
				Laboratory-based
Cell-free DNA	HMP	Correlated with posttransplant serum creatinine, creatinine clearance, eGFR, and DGF^24^	Exploratory	Advantages
				Detectable in HMP perfusate
				Also used in liver and lung machine perfusion
				Disadvantages
				Laboratory-based
Histones	HMP	Associated with DGF and PNF, 1-y survival, and 1-y graft failure risk^25^	Exploratory	Advantages
				Detectable during HMP (most frequently used MP modality)
				Disadvantages
				Laboratory-based
				Exploratory, limited literature available
MicroRNA	HMP	Associated with DGF and eGFR^26-28^	Exploratory	Advantages
				High specificity potential
				Detectable in HMP perfusate
				Disadvantages
				Laboratory-based
Heme/hemoglobin	NMP	Kidneys exposed to high-free hemoglobin failed during NMP and resulted in ferroptosis^29,30^	Exploratory	Advantages
				Provides mechanistic insight in inflammatory injury
				Disadvantages
				NMP only
				Laboratory-based
				Only described for DCD and discarded kidneys
Metalloproteinase	HMP	Perfusate MMP-2 and MMP-9 are associated with DGF^31^	Exploratory	Advantages
				Reflect matrix remodeling injury
				Disadvantages
				Laboratory-based
				Very limited evidence base
Metabolites	HMP	Lower glucose, inosine, and gluconate, and higher leucine and lactate for DGF kidneys^32^	Exploratory	Advantages
		Metabolites associated with mitochondrial function are associated with death-censored graft failure^33^		
				Real-time metabolic profiling possible
				Broad mechanistic insight
				Disadvantages
				Requires a metabolomics platform
				Complex data interpretation
				Kidneys are metabolically less active during hypothermia

Clinical studies were defined as studies in which the parameter/(bio)marker was tested for transplanted kidneys. Preclinical studies were defined as studies in which the parameter/(bio)marker was tested on nonused human kidneys or animal kidneys. Explorative studies were defined as hypothesis-generating studies.

DCD, donation after circulatory death; DGF, delayed graft function; eGFR, estimated glomerular filtration rate; EPO, erythropoietin; FENa, fractional excretion of sodium; HMP, hypothermic machine perfusion; IGF, immediate graft function; IL-18, interleukin-18; LSCI, laser speckle contrast imaging; MMP, matrix metalloproteinase; MP, machine perfusion; NGAL, neutrophil gelatinase-associated lipocalin; NMP, normothermic machine perfusion; PNF, primary nonfunction; RBF, renal blood flow.

## URINARY AND PERFUSATE BIOMARKERS

Combining the measurement of hemodynamic parameters with noninvasive analysis of biomarkers offers a promising strategy to assess kidney quality and improve donor utilization rates. Whereas hemodynamic parameters primarily reflect the vascular compartment, urinary and perfusate biomarkers provide information on functional, immune, and vascular aspects of the graft. Consequently, analysis of biomarkers in the circulating perfusate and urine offers a more comprehensive and multidimensional assessment of kidney graft quality.

Lactate dehydrogenase, glutathione S-transferase, and NGAL have emerged as potential biomarkers associated with posttransplant kidney function. However, none of the investigated markers is currently routinely used during pretransplant kidney assessment as they have limited positive predictive value and sensitivity. Therefore, we further focus on other emerging biomarkers.

### Functional Markers

Kidney functional metrics such as urine production, glomerular filtration, and tubular absorption can be measured during perfusion. Urine output is a commonly studied functional marker during NMP. It is incorporated in the quality assessment score (QAS), which also includes RBF and macroscopic assessment during perfusion to score kidneys pretransplant.^34^ Higher urine output has been linked to less injury^18^ and IGF (numerically only).^10^ Perhaps contra-intuitively, it has been demonstrated that proteinuria during NMP correlates with improved eGFR at 3 mo posttransplant.^10^ Two animal studies also reported ex situ proteinuria; however, they did not find a correlation between urinary protein levels and kidney function (sodium excretion, creatinine clearance, oxygen consumption, and urine output) during NMP.^35,36^ FENa, a marker of tubular function, can be measured in the circulating perfusate, and research has shown that well-performing kidneys, assessed by global appearance, blood flow, and urine output, excrete less sodium than poor performers,^6^ although others did not observe differential excretion.^10^

Focusing on glomerular filtration, creatinine clearance is another critical functional parameter to determine kidney quality. In a study including 56 discarded deceased donor kidneys, Hosgood and Nicholson^18^ reported a significantly greater decrease in perfusate creatinine for kidneys with a favorable QAS compared with kidneys with QAS ≥4. Kidneys with better creatinine clearance during NMP also showed significantly higher RBF and urine output, which are associated with better grafts.^18^ In line with these findings, another study also showed higher creatinine clearance during perfusion among good performing kidneys.^6^

Kidneys act as an endocrine organ, secreting (pro)renin, erythropoietin (EPO), 1,25-dihydroxy vitamin D, and urodilatin. Lin et al^20^ demonstrated that human kidneys release (pro)renin, EPO and active vitamin D in the perfusate, and urodilatin in the urine during NMP. In contrast, these hormones were significantly lower or undetectable in HMP perfusates, likely reflecting the reduced metabolic activity under hypothermic conditions. The authors correlated hormone release with posttransplant outcomes, reporting that kidneys with IGF have higher urinary urodilatin levels during NMP. Furthermore, urodilatin levels inversely correlated with DGF duration, and greater EPO release was associated with higher serum creatinine levels on day 7 posttransplant.

Overall, these studies suggest that functional markers measured during machine perfusion are associated with posttransplant graft function. Proteinuria, FENa, clearance measurements, and endocrine biomarkers each provide complementary information on different aspects of kidney physiology, including glomerular barrier integrity, tubular function, and glomerular filtration (Table 1). While these markers offer valuable functional insights, most are currently limited to NMP, require laboratory-based analysis, and cannot be measured continuously in real-time. In addition, some biomarkers lack validated thresholds for clinical decision-making (Table 1).

### Cytokines and Complement Activation

Various immunologic mediators are released by the kidney in response to the pathophysiologic effects of deceased organ donation and ischemia. These mediators are likewise released during machine perfusion, where they may serve as indicators for graft injury and immunogenicity before transplantation, contributing to kidney graft quality assessment. We showed that various cytokine concentrations increase during 2-h clinical NMP, including tumor necrosis factor, interleukin (IL)-6, IL-10, and IL-18.^37^ In contrast to another study,^23^ we did not observe a more pro-inflammatory milieu, but rather a mixed one. Furthermore, we did not observe a correlation between differential cytokine release across and short-term posttransplant outcomes, including DGF and acute rejection. Interestingly, Dumbill et al^10^ reported significantly higher perfusate IL-18 at 2 h NMP for kidneys that displayed DGF compared with IGF kidneys.

Other immunological mediators include complement cascade factors, which contribute to allograft injury, including ischemia-reperfusion injury. A recent study reported complement factors C3a, C3d, and C5b-9 (terminal complement activation) in perfusate samples of porcine and human kidneys undergoing NMP.^38^ In their porcine model, the authors also demonstrated a significant release of pro-inflammatory cytokines IL-6, IL-8, and tumor necrosis factor in the perfusate, which all correlated with perfusate C3a and C5b-9.^23^ Interestingly, porcine kidneys with above median (105 AU/mL) perfusate C5b-9 levels showed significantly impaired creatinine clearance during NMP,^23^ likely reflecting decreased graft function. Fallon et al^22^ investigated complement activation and its effect on renal function in a clinical setting. They observed that elevated perfusate C3b and C5b-9 levels were associated with DGF and an increased risk of prolonged DGF. Similar results were observed during HMP, with elevated perfusate C3 levels and enrichment of the complement cascade.^21^ Notably, perfusate complement levels were inversely correlated with 12-mo eGFR.^21^

### Immune Cells

Machine perfusion allows to investigate the release of donor kidney immune cells into the perfusate. Consequently, quantifying tissue-resident immune cells (TRLs) in the perfusate may serve as a noninvasive biomarker of graft quality because their presence likely reflects underlying tissue injury, inflammation, or immune activation within the kidney. The efflux of immune cells from the kidney into the perfusion circuit was already reported for porcine kidneys, which released helper-, cytotoxic double-negative-, and CD4^+^CD8^+^-T cells.^39^ Besides the various T-cell populations, B cells, basophils, eosinophils, and natural killer cells were also detected in NMP perfusates. Until recently, the clinical impact of perfusate TRLs remained unclear. Analysis of clinical HMP and NMP perfusates showed, in line with Stone et al,^39^ a heterogeneous population comprising CD4^+^ T cells, CD8^+^ memory T cells, natural killer T cells, memory T cells, and helper-like innate lymphoid cells, but not B cells.^38^ Furthermore, TRL release was not associated with donor characteristics, perfusion parameters, damage-associated cell-free DNA (cfDNA) and NGAL, and, most importantly, posttransplant outcomes.^38^

Although research on immune cells in perfusate remains limited, transcriptomic profiling of kidney tissue during NMP provides insight into the kidney’s cellular landscape under machine perfusion. Bulk RNA sequencing revealed that kidneys that developed DGF exhibited enrichment of pro-inflammatory and immune-related pathways, including those involved in leukocyte recruitment, migration, and macrophage activation.^40^ Another recent study by our group using single-nucleus RNA sequencing confirmed the presence of immune cells in the kidney but found that their abundance and gene expression profiles remained largely unchanged during NMP.^41^ Notably, neither study linked these transcriptomic findings to surrogate biomarkers in the perfusate, leaving a gap in our understanding of how tissue-level immune activity is reflected in perfusate markers.

Insights from single-cell RNA sequencing of human liver allografts during NMP may be informative in this context. Hautz et al^42^ showed that liver macrophages, despite stable abundance, underwent significant transcriptional reprogramming toward anti-inflammatory and tolerogenic phenotypes. Concurrently, macrophages released into the perfusate were major sources of IL-6, with significantly higher IL-6 expression and perfusate concentrations in discarded livers compared with transplanted ones, linking macrophage-derived cytokines to graft injury. By analogy, profiling macrophage activation in kidney perfusate could provide a dynamic, noninvasive readout of tissue-level immune activity and graft quality. This association has not yet been fully explored. Yet, in our study, perfusate IL-6 did not associate with posttransplant graft function.^37^

These markers provide mechanistic insight into inflammatory injury and can be measured simultaneously using multiplex assays, enabling broad profiling of the graft inflammatory response. However, their assessment relies on laboratory-based analysis of perfusate samples, precluding real-time evaluation during machine perfusion. Furthermore, their application remains exploratory, with limited standardization (Table 1).

### Damage-associated Molecular Patterns

Upon injury, necrosis, and apoptosis, cells release cfDNA, which are circulating, short fragments of DNA. Prior research has shown that perfusate cfDNA is a promising predictor of graft viability and outcomes in lung and liver machine perfusion,^43,44^ sparking interest in its application during kidney machine perfusion. Although it is already established that kidneys release cfDNA during machine perfusion,^38^ its correlation to posttransplant outcomes is still under investigation. It was recently shown that perfusate cfDNA was higher during HMP for kidneys with lower creatinine clearance on all posttransplant days, lower day 3 eGFR, elevated days 3 and 4 serum creatinine, and the occurrence of DGF.^24^ Moreover, the authors reported a negative correlation between perfusate cfDNA levels and kidneys with a Kidney Donor Profile Index ≥70%. Besides, cfDNA levels from high Kidney Donor Profile Index donors were negatively associated with creatinine clearance and eGFR on day 3 posttransplant.^24^ Along with cfDNA, extracellular histones are released upon cell stress and death, which could be used as a biomarker to assess kidney quality. In a cohort of 390 kidneys preserved by HMP, significantly higher histone levels were reported in perfusate for DGF and primary nonfunction (PNF) kidneys compared with IGF, with no difference between DGF and PNF.^25^ Further analyses revealed that perfusate histone H3 concentration was an independent risk factor for DGF in both univariable and multivariable analysis. However, the predictive value was low (area under the curve [AUC] = 0.674). In addition, kidneys with histone H3 levels <0.70 µg/mL had a significantly better 1-y survival, continuing up to 5 y posttransplant. In addition, univariable and multivariable regression showed that 1-y graft failure risk increased with increasing perfusate histone concentrations.^25^

Upon donor death and ischemia, neutrophils release neutrophil extracellular traps (NETs), web-like structures containing DNA, histones, neutrophil granular enzymes, and matrix metalloproteinases (MMPs). In a postischemic acute kidney injury mouse model, which resulted in tubular necrosis, local formation of NETs was accompanied by increased expression of inflammatory and injury-related genes.^45^ In a postischemic acute kidney injury mouse model, which resulted in tubular necrosis, local formation of NETs accompanied by increased expression of inflammatory and injury-related genes.^45^ It was proposed that cytotoxic NETs lead to more cell necrosis, further exacerbating acute renal injury. Although NETs have not been investigated in kidney perfusates, they have been detected in ex situ lung perfusates and were associated with recipient clinical outcomes, including days on the ventilator and length of stay in the intensive care unit.^46^

MicroRNAs (miRNAs) regulate gene expression, thereby modulating (patho)physiologic processes, with several implicated in kidney pathophysiology. Several miRNAs were identified in perfusate and associated with DGF or eGFR: miRNA-18a, -20a, -21, -142-5p, -144-3p, -144-5p, -223-3p, -363-3p, -454-3p, -486-5p, and -502-3.^26,27^ Rather than free miRNA, extracellular vesicle-bound miR-2018-5p during HMP was also elevated for DGF kidneys and inversely correlated with eGFR.^28^

Overall, these markers of cellular injury (cfDNA, histones, and miRNAs) have emerged as promising biomarkers during machine perfusion. A key advantage is that they can be detected in HMP perfusate, the most commonly used machine perfusion modality. However, their assessment requires laboratory-based analysis and is therefore not available in real-time. Moreover, their application remains exploratory, with limited validation to support routine use (Table 1).

### Hemeproteins and MMPs

NMP requires an oxygen carrier in the perfusate for optimal oxygen delivery to the graft, preventing warm ischemic damage. Therefore, matched packed red blood cells (RBCs) are often used in an NMP circuit. However, mechanical perfusion with RBCs results in hemolysis and the free heme proteins expose the kidney to the potentially harmful effects of heme (the nonprotein group in hemoglobin) and iron.^29^ Perfusate free heme levels are indeed significantly increased during NMP, although no correlation with functional parameters, such as urine output, oxygen consumption, RBF, and urinary NGAL, was found.^30^ In a separate cohort of discarded kidneys, free heme levels did not correlate with increased expression of genes associated with apoptosis, inflammatory cytokines, chemokines, and oxidative stress.^30^ These results suggest that free heme does not have detrimental effects on the graft during NMP. de Haan et al^29^ reported progressive hemolysis and subsequent buildup of free hemoglobin in the perfusate during NMP of discarded kidneys. In contrast to the findings of Hosgood et al,^30^ they reported that kidneys exposed to the highest levels of free hemoglobin failed during NMP. However, it should be noted that perfusion times were significantly different between both studies. Moreover, RBC-based NMP led to increased iron uptake and storage, resulting in iron accumulation within the kidney tissue. The authors further demonstrated evidence of iron-dependent cell death (ie, ferroptosis), indicated by elevated levels of phospholipid peroxidation byproducts.^29^ As cell death further damages the allograft, measuring markers that serve as a proxy for ferroptosis, such as malondialdehyde and 4-hydroxynonenal,^29^ may offer insight into kidney quality.

Besides the induction of ferroptosis, hemoglobin-induced oxidative stress also contributes to activation of MMPs. Specifically, studies showed that MMP-2 and MMP-9 are involved in acute and chronic kidney injury, tubular atrophy, fibrosis, and kidney failure.^47^ During kidney HMP, both MMP-2 and MMP-9 were detectable in perfusates and were significantly associated with DGF. In contrast, the commonly used kidney injury markers NGAL and lactate dehydrogenase did not differ between DGF and IGF kidneys.^31^ Despite this added predictive value, MMPs have not been investigated further as biomarkers during kidney machine perfusion.

Heme/hemoglobin and MMPs represent exploratory biomarkers with mechanistic rather than clinical validation to date. Both markers are currently limited by laboratory-based assessment, and their translation into clinically applicable, real-time decision-making tools during perfusion remains to be established (Table 1).

### Metabolites

The perfusate composition changes during machine perfusion because of nutrient uptake and other cellular metabolic processes in the kidney. Metabolic profiling of the perfusate may reflect pathophysiological changes in the kidney indicative of graft quality and viability. During nonoxygenated HMP, 28 metabolites were detected, 6 of which originated from the preservation fluid.^32^ Of these 28 metabolites, glucose, inosine, and gluconate were significantly lower in DGF kidneys (AUC 0.842, 0.833, and 0.851, respectively), while leucine and lactate levels were elevated during perfusion.^32^ These metabolic changes are in line with previous findings that DGF grafts fail to reestablish aerobic respiration after oxygenation, showing continued ATP catabolism and lactate release.^48^ Contrarily, another study reported unchanged glucose levels in the perfusate during HMP, yet lactate levels increased.^49^ Furthermore, they did not report a correlation between metabolite concentrations and posttransplant graft function or tubular transporter damage. A larger, multicenter study on perfusate metabolites revealed that 6 metabolites were associated with death-censored graft failure during a median follow-up of 5 y: α-ketoglutarate (AKG), 1-palmitoyl-2-docosahexaenoyl-glycerophosphocholine (-GPC), 1-palmitoyl-2-dihomo-linolenoyl-GPC, 1-stearoyl-2-docosahexaenoyl-GPC, 1-carboxyethylphenylalanine, and 3-carboxy-4-methyl-5-propyl-2-furanpropanoate (CMPF).^33^ Additionally, from these 6 metabolites, 4 were also associated with DGF: CMPF, AKG, 1-stearoyl-2-docosahexaenoyl-GPC, and 1-palmitoyl-2-docosahexaenoyl- GPC. Interestingly, all metabolites of interest are associated with mitochondrial function. AKG, a key intermediate in the tricarboxylic acid cycle, potentially reduces proximal tubule injury, thereby reflecting protective effects against oxidative stress. While the 3 -GPCs can reduce hypoxia-induced production of reactive oxygen species by preserving complex I function, increased extracellular -GPC concentrations may also reflect ischemia-induced necrosis. Similarly, CMPF can induce proximal tubular cell damage and inhibit mitochondrial respiration.

Another marker associated with aerobic respiration is flavin mononucleotide (FMN), a small molecule bound to mitochondrial complex I, which dissociates upon reperfusion, resulting in reduced complex I activity and consequent mitochondrial dysfunction. A study using discarded kidneys reported >2-fold increased perfusate FMN levels after 1 h of NMP, which did not correlate with the QAS.^17^ Notably, 7 of 11 kidneys were ultimately transplanted; among these, 1 developed DGF and 2 experienced PNF. Interestingly, these 3 kidneys showed higher FMN levels compared with IGF kidneys. Additionally, perfusate FMN was strongly correlated with serum creatinine at days 14, 30, and 90 posttransplant.^17^ In contrast, a larger trial found that FMN levels during HMP were not clinically relevant, regardless of whether oxygen was supplied during perfusion.^50^ Notably, validation of perfusate FMN in a subset of the cohort by liquid chromatography with tandem mass spectrometry revealed that FMN was detectable in only 1 sample, leaving the clinical relevance of FMN uncertain. It should be noted, however, that FMN is released upon reverse electron transfer, a process that is suppressed under hypothermic conditions.

While real-time metabolic profiling is feasible, its clinical application is limited by the need for specialized metabolomics platforms and the complexity of data interpretation. Furthermore, the relatively low metabolic activity of kidneys during HMP may limit the power of metabolite-based signals in this setting (Table 1).

In summary, a range of urinary (urine output, clearance, and hormones) and perfusate (immune-related, damage-associated, and ferroptosis-derived) markers can provide insight into different compartments of the kidney during machine perfusion. While some are associated with posttransplant outcomes (Table 1), a biomarker directly and reliably predictive of clinical results has not yet been identified.

## LIMITATIONS AND FUTURE PROSPECTIVES

Functional assessment of donor kidneys and perfusate and urinary biomarkers during kidney machine perfusion are of major interest for the prediction of posttransplant outcome. Despite the plenitude of studies aimed at identifying noninvasive biomarkers and measurements, none of them have yet been implemented in routine quality assessments. Many of the markers described in this review originate from single center studies, often with a small sample size, limiting the generalizability and robustness of the results. Furthermore, there is great methodological heterogeneity across studies, reflected by the variation in circulatory and brain death donors as well as the perfusion modality (oxygenated or non-oxygenated HMP and NMP), perfusion time, and use of transplanted or discarded grafts. Studies using discarded grafts offer limited predictive insight, as posttransplant outcomes are not available. A similar variation is observed for the reported outcome measures. Although most studies include DGF as an endpoint, the reported incidence varies widely, from about 10% to >80%, which limits the consistency, interpretation, and generalizability of the findings. Interestingly, biomarker concentrations were often similar between DGF and PNF kidneys, or PNF was not included as a study endpoint. However, the inclusion of PNF kidneys is crucial, as it represents 1 of the most severe short-term outcomes. Identifying such kidneys before transplantation could prevent the transplantation of nonfunctional grafts and recipient immunization and retransplantation. Other transplant outcomes, such as serum creatinine, eGFR, graft failure, and rejection, were also inconsistently represented across studies. Consequently, many biomarkers were evaluated for only a specific outcome, again limiting robustness of the conclusions and making it difficult to compare biomarker performance across studies. Although biomarker research faces similar barriers across organs, the slower progress in kidney machine perfusion may also be explained by the complex physiology and high metabolic demands of the kidney, as comparable biomarkers appear more promising in liver perfusion research.

Translating biomarkers from the preclinical research stage into clinical practice remains challenging, owing to the complex and multifactorial nature of transplant-related injuries and the limited availability of well-designed cohort studies. Therefore, future studies require larger sample sizes, best achieved through multicenter cohort designs, either retrospective or prospective. Such an approach would improve the reliability of biomarker discovery and improve risk assessment by using standardized study endpoints. Equally important is the implementation of a consistent sampling protocol, ideally after at least 1 h of perfusion, allowing sufficient time for complete cortical perfusion^15^ and ensuring that biomarkers reflect the kidney’s functionally active compartment. Furthermore, future studies should aim to translate tissue-level signatures, such as those identified by transcriptomic profiling, into measurable, noninvasive perfusate biomarkers. In parallel, integrating complementary biochemical markers of injury, such as ferroptosis-related lipid peroxidation markers (eg, malondialdehyde, 4-hydroxynonenal), and mitochondrial-derived metabolites, may provide a broader assessment of kidney graft quality beyond immune activation alone.

Collectively, these studies highlight that patterns of immune activation, cellular responses, and metabolic alterations—particularly those related to mitochondrial function and anaerobic metabolism—play a key role in determining kidney performance after transplantation. Accordingly, alongside hemodynamic measurements, analysis of perfusate or urinary markers reflecting these processes can provide valuable insight into graft viability and may aid in predicting posttransplant outcomes.
